# The Impact of Parents’ Subjective Preparedness on Their Children’s Post-Traumatic Symptoms Following Surgery

**DOI:** 10.3390/children11070780

**Published:** 2024-06-27

**Authors:** Fortu Benarroch, Rony Kapel Lev-Ari, Amichai Ben-Ari

**Affiliations:** 1 Herman Dana Division of Child and Adolescent Psychiatry, Hadassah-Hebrew University Medical Center, Jerusalem 9112001, Israel; amichaiba@ariel.ac.il; 2 The Faculty of Medicine, The Hebrew University of Jerusalem, Jerusalem 9112001, Israel; 3 Department of Behavioral Sciences, Ariel University, Ariel 40700, Israel; rony.kapellev@msmail.ariel.ac.il

**Keywords:** traumatic stress, prevention, children, pediatric surgery, preparation

## Abstract

The role of parental factors in the emergence of post-traumatic stress symptoms (PTSSs) following pediatric surgeries is well recognized, but the specific influence of parents’ subjective preparedness for their child’s surgery has not been explored. In a study involving 253 children hospitalized in a pediatric surgery ward, parents completed a demographic questionnaire during their child’s stay, which included the question, “As a parent, have you been prepared for the surgical intervention your child is undergoing?” Four months post-surgery, the same parents were interviewed using two questionnaires that evaluated their children’s post-traumatic symptoms. Our findings indicate that in emergency surgical settings, children whose parents felt prepared experienced significantly fewer PTSSs compared to children whose parents did not feel prepared. In contrast, for elective surgeries, parental subjective perception of preparedness did not significantly impact the children’s PTSSs. We conclude that for emergency surgical procedures, addressing parents’ subjective preparedness could be crucial. Further research is necessary to develop targeted interventions that leverage this insight to minimize the risk of PTSSs in children undergoing emergency surgeries.

## 1. Introduction

During hospitalization in a pediatric surgery ward, children may be exposed to potentially traumatic experiences resulting from invasive procedures, pain, uncertainty, and even life-threatening conditions. Consequently, some children may develop a post-traumatic stress response. Although most children will recover, we found in a previous study that 10.39% scored above the cutoff for Post-Traumatic Stress Disorder (PTSD) on validated questionnaires and 17.1% had at least one symptom in each of the three classical post-traumatic symptoms’ clusters (re-experiencing, avoidance, and hyper-arousal) [1]. This was in line with similar studies in the field [2,3]. Since a significant number of children experience PTSSs without meeting the full criteria for PTSD, yet still suffer from emotional distress and/or functional impairment, the National Child Traumatic Stress Network has named this condition Pediatric Medical Traumatic Stress (PMTS), which is essentially an abbreviation for partial PTSD resulting from medical stress [2]. This condition can disrupt various aspects of a child’s life, including the development of medical phobia that compromise recovery due to non-compliance with medical follow-up [2]. Consequently, there is considerable interest in identifying factors that influence whether a child recovers or develops PMTS after surgery.

Previous research has consistently demonstrated that parental attitudes and behaviors significantly affect children’s experiences of medical procedures and their recovery outcomes [4,5,6,7]. This influence is particularly critical in young children, who can only receive interventions mediated through their parents. Behavioral preoperative preparation has been advocated in the psychological and medical literature as a way to reduce children’s preoperative anxiety and facilitate post procedure recovery. An estimated 78% of all major hospitals offer such programs to children and their parents [8]. These preparation programs provide narrative information, an orientation tour, teaching of coping and relaxation skills to children and their parents and may be effective in reducing child and parent anxiety [9]. We are child psychologists and psychiatrists serving as consultants to the pediatric surgery department at a tertiary medical center. We have observed a lack of awareness regarding PMTS and have initiated a project aimed at developing a tool for identifying children at risk for PMTS. In a previous study, we showed that parental beliefs and levels of distress significantly contribute to the development of post-traumatic stress symptoms (PTSSs) in children, suggesting that managing parental stress and beliefs could mitigate the risk of PMTS [10]. However, in emergency surgery scenarios, there is no opportunity to engage with parents preoperatively regarding their beliefs, and their stress levels are invariably high due to the emergency circumstances. To reduce the risk of PMTS under these conditions, we must address other contributing factors. Through our fieldwork, we have observed great variability in the subjective perception of preparedness of parents who arrive at the hospital under emergency conditions. Those parents cannot be a part of standard preoperative psychological preparation programs. This led us to explore whether parents’ subjective perception of their readiness for their child’s surgery might play a role in the child’s post-surgical outcomes, specifically in terms of PTSSs. This study aims to assess the relationship between parents’ perceived preparedness and the development of PTSSs in their children following surgical procedures. Our research hypothesis was that the subjective perception of parents regarding their preparedness for their child’s surgery would significantly influence the development of PTSSs in their children.

## 2. Materials and Methods

This research report is based on data extracted from our abovementioned project on PMTS, which is still under study. It was conducted after the approval of the Helsinki Committee at the Hadassah Medical Center in Jerusalem [0437-14-HMO]. The inclusion criteria were children aged one to six years who were hospitalized in the pediatric surgery department and whose parents consented voluntarily to participate in the study. The exclusion criteria were any type of head injury. All the parents signed an informed consent form. Parents of 285 children were approached. A total of 32 subjects dropped out during the study due to unwillingness and lack of time.

Participants: The final sample consisted of 253 children. A total of 116 children endured a simple surgery (e.g., tonsillectomy, hernioplasty) (46.8%), 21 children endured a surgical procedure not performed in the operating room (8.5%), 15 (5.9%) had surgeries dealing with burns, and the remaining 101 had other types of surgeries with medium complexity (39.9%).

Furthermore, of participants, 146 (57.7%) were boys and the remaining 107 (42.3%) were girls. A total of 152 were Jews and 101 were Arabs. In most cases, the mother of the child filled out the questionnaires (n = 177, 70%), while in the remaining cases, it was the father (n= 76, 30%). Children’s ages ranged between 1 and 6, with the average age being 2.99 years (sd = 1.55). The mothers’ ages ranged between 19 and 54, with an average of 31.77 (sd = 6.54). The fathers’ ages ranged between 21 and 70, with an average of 34.92 (sd = 8.03). The children had between 1 and 13 siblings, with an average of 3.53 siblings (sd = 2.08). Fathers had an average of 10.08 years of education (sd = 6.38) and mothers had an average of 9.99 years of education (sd = 5.98). Children had an average of 3.83 (sd = 1.08) rooms in their household, this variable giving a better understanding regarding the family’s socioeconomic status. Furthermore, children lived in households that had an average of 5.56 (sd = 2.08) family members living with them.

We categorized the hospitalization as ‘emergency’ when the patient was unexpectedly admitted to the emergency department due to an acute condition and subsequently transferred for surgery. By ‘elective’ surgery, we refer to situations where the child was referred for a planned surgery following a routine evaluation in the clinic. Overall, 174 (68.8%) of the children underwent elective surgery and the remaining 78 (31.2%) underwent emergency surgery. The duration of the hospitalization ranged from 1 to 37 days (M = 4.71, SD = 5.70). Although it is a convenience sample, demographic and hospitalization data of the group were similar to those of the overall population in the same pediatric surgery ward between the ages of 1 to 6 (N = 6231) during the same year. Consequently, it can be assumed that the sample represents the general population of those hospitalized in the ward for that year.

### 2.1. Measures

1. Demographic questionnaire: Demographic variables included age and gender of the child and participating parent, social group (Arab or Jew), number of siblings, years of education of the parent, number of rooms in the household and a yes/no question: “As a parent, have you been prepared for the surgical intervention your child is undergoing?”
2. The UCLA PTSD Reaction Index for DSM-5, Parent/Caregiver Version for children aged six and below [11], is a questionnaire designed to evaluate post-traumatic stress in young children. Parents complete this survey, which is known for its robust reliability and validity, showing Cronbach’s alpha scores between 0.88 and 0.91. This tool does not exhibit notable discrepancies in outcomes across various ethnic or religious groups. Its results align with those from established PTSD assessments like the PTSD Checklist, PTSD Symptom Scale, and Harvard Trauma Questionnaire.
3. The Young Child PTSD Checklist (YCPC) [12] serves as a self-report instrument to gauge PTSD in toddlers and preschool-aged children (0–6 years). Parents report using this checklist, which demonstrates high reliability, with Cronbach’s alpha scores of 0.77 for arousal, 0.79 for avoidance, and 0.81 for reliving, validating its diagnostic accuracy for early childhood PTSD.

These two questionnaires were initially translated from English to Hebrew by the researchers, then back-translated to English by two English-speaking reviewers, and subsequently back-translated to Hebrew by two different reviewers. Where inconsistencies arose, an additional reviewer, a native English speaker with expertise in translation, determined the final phrasing. The same procedure was applied for the Arabic translation, with assistance from a native Arabic-speaking psychologist holding a master’s degree. Cronbach’s alpha values for the CBCL in our sample were as follows: Hebrew version 0.981, Arabic version 0.791; for the YCPC: Hebrew version 0.986, Arabic version 0.894.

### 2.2. Procedure

The research procedure consisted of two stages. (1) During the hospitalization in the pediatric surgery ward, an explanation of the study was provided to parents, who signed an informed consent form and completed the demographic questionnaire. (2) In the second phase, four months after hospitalization, parents completed the UCLA-PTSD and YCPC questionnaires during a face-to-face interview with psychology master’s degree students who were specially trained for this purpose.

### 2.3. Data Analysis

In order to test our hypothesis, we initially conducted two sets of hierarchical regression. In the first model, the dependent variable was the YCPC variable, and in the second, it was the UCLA-PTSD variable. In both models, we entered the independent variables in three steps. In the first step, we added the covariant descriptive variables—child’s gender; age; parent’s gender, education years and age; number of siblings; number of rooms in the household (i.e., in an attempt to determine the family’s socio-economic status). In the second step, we added the type of surgery variable (emergency/elective). Finally, in the third step, we added the parent’s subjective state of preparation for the surgery (prepared/unprepared).

In the next step, we used the cutoff for PTSD in both the YCPC and the UCLA-PTSD questionnaires and compared the chance of a child to meet the cutoff for PTSD dependent on their parent’s subjective feeling of being prepared or unprepared for the surgery. In order to answer this hypothesis, we conducted a crosstab examination using the PTSD (Yes/No) variables and the parent’s preparation variable (prepared/unprepared).

In our final hypothesis, we wanted to see whether the parents’ subjective feeling of being prepared affected differently regarding children who had elective versus emergency surgery.

In order to test this, we used multivariate analysis. The dependent variables were the UCLA-PTSD and YCPC total scores, and the fixed factors were surgery type (elective/emergency) and parent’s preparation (prepared/unprepared). We examined both the main effects of each variable and the interaction effect regarding their association with the dependent variables.

## 3. Results

We found that the percentage of children scoring above the threshold on PTSD questionnaires four months after hospitalization was 10.1% in the UCLA PTSD and 11.3% in the YCPC. The regression results showed the model is significant, explaining 43% of the variance in UCLA-PTSD and 30.4% of the variance regarding YCPC. Furthermore, both surgery type and parent’s subjective feeling of being prepared were significant predictors of both YCPC and UCLA-PTSD: adding them to the model, while keeping constant all the other demographic variables, yielded a significant change in the variance. Out of the demographic variables, only the child’s gender, the mother’s education years, and the number of rooms in the house were significant predictors of the child’s score in the PTSD questionnaires. The results are presented in Table 1.

In the next analysis, we ran a crosstab to demonstrate the manner in which the parents’ feeling that they were prepared influenced the children’s chance for meeting the PTSD cutoff score in each of the PTSD questionnaires.

The results are shown in Table 2.

In order to gain a better understanding of these associations, we also conducted two independent *t*-tests. We added the UCLA-PTSD and the YCPC total scores as the dependent variables and the parents’ feeling of preparation (yes or no) as the control variables. The results showed that children whose parents felt that they were not prepared for the surgery showed significantly higher scores after the medical procedure (UCLA-PTSD (t(190) = 2.202, *p* < 0.05); YCPC t(190) = 3.578, *p* < 0.001).

In the next step, we wanted to see whether the parents’ subjective feeling of being prepared affects differently those children who had elective versus emergency surgery.

The results were analyzed using multivariate analysis. The dependent variables were the UCLA-PTSD and YCPC total scores, and the fixed factors were surgery type (elective/emergency) and parent’s preparation (Yes/No).

**Figure 1:**
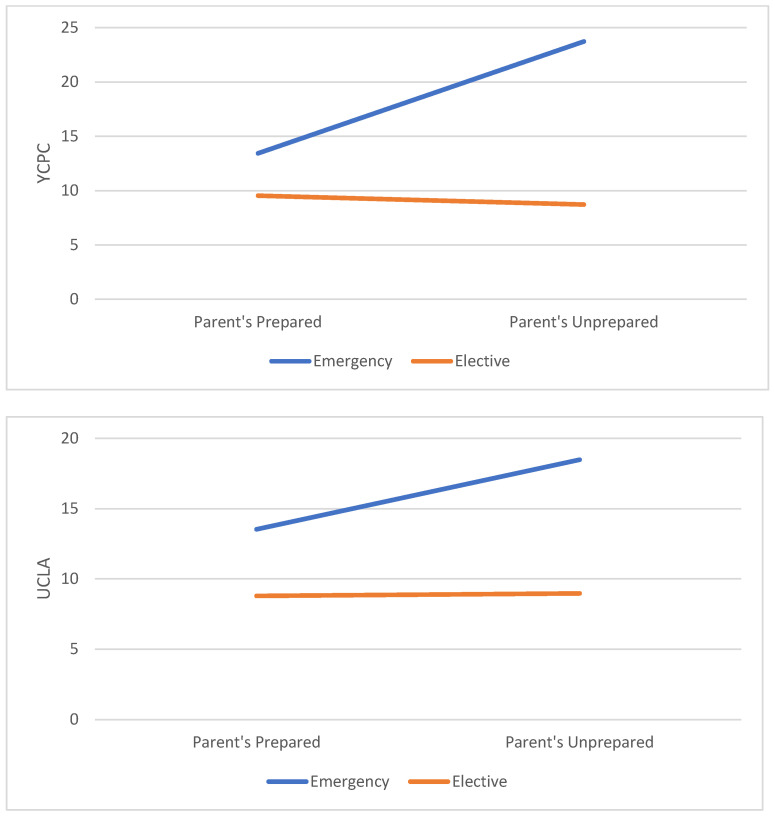
A graphical demonstration of the impact of parents’ subjective perception of preparedness on the total scores in the PTSS questionnaires.

The results showed significant effects for the surgery type (Roy’s Largest Root = 0.056, F(2.185) = 5.176, *p* < 0.01, η_p_^2^ = 0.051) and for parent’s feelings of being prepared (Roy’s Largest Root = 0.032, F(2.185) = 2.999, *p* < 0.05, η_p_^2^ = 0.029). Additionally, a significant interaction was found (Roy’s Largest Root = 0.068, F(2.185) = 6.251, *p* < 0.01, η_p_^2^ = 0.061), showing that only children undergoing emergency surgery, were positively affected by their parents’ feeling of being prepared. The results are shown in Figure 1.

## 4. Discussion

This study is the first to examine the association between parents’ subjective perception of their preparedness for their child’s surgery and the development of the child’s post-traumatic stress symptoms (PTSSs). This relationship was analyzed in the context of both elective and emergency surgeries. We found that the percentage of children scoring above the threshold on PTSD questionnaires four months after hospitalization was around 10–11%, a finding very similar to reports from other groups in the literature [1,2,3].

Hierarchical regression analysis, controlling for other demographic variables, revealed that including parental preparedness significantly increased the explanatory power of our model. Cross-tabulation results indicated that parents who felt prepared at the time of hospitalization had children who were significantly less likely to have total scores beyond the cutoff for PTSD four months later. When PTSS scores from questionnaires were used as a continuous measure, the results were also significant and in the same direction.

The multivariate analysis demonstrated that in emergency surgical contexts, children of parents who felt prepared experienced significantly fewer PTSSs compared to those whose parents felt unprepared. In contrast, this factor did not significantly influence PTSSs in elective surgeries.

Our literature review found no previous studies that specifically address the impact of parents’ subjective preparedness on surgical outcomes. Notably, most research on PTSS prevention post-surgery has focused on elective procedures [6,8,9,13], with no comparative studies on emergency versus elective surgeries regarding PTSSs.

A potential claim could be that the parents’ response to whether they felt prepared might actually serve as a proxy for parental distress. Nevertheless, given that parental distress significantly influences child distress, if the question about preparedness indeed acted as a proxy, a significant effect would also be expected in elective surgeries.

### Limitations

One limitation arises from parental reporting of their child’s symptoms. Parents feeling unprepared may overestimate their child’s PTSSs, influenced by their own stress levels, as indicated by published studies [14,15]. However, PTSS assessments in children aged 1–6 years have to rely mainly on parental reports. An additional limitation is the relatively small sample size, which only represents the population of this specific medical center. While the sample is multicultural, the issue warrants further research involving different centers to examine the generalizability of the findings.

## 5. Conclusions

Our findings suggest that interventions aimed at preventing PTSSs in children undergoing surgical procedures should be tailored and tested distinctly for elective versus emergency surgical contexts. A key finding relates to very young children undergoing emergency surgeries; those whose parents felt prepared experienced fewer PTSSs. This underscores the importance of addressing parental perception of preparedness in emergency surgical situations. Future research should explore the factors that can help parents feel more adequately prepared for their child’s emergency surgery. Such insights would facilitate targeted training for medical and nursing staff in pediatric surgery departments on effective approaches to support these parents, potentially reducing the risk of PMTS.

## Figures and Tables

**Table 1:** Hierarchical regressions predicted YCPC and UCLA-PTSD total scores.

Variables	YCPC				UCLA			
	B	Std Error	β	R^2^ Change	B	Std Error	β	R^2^ Change
Step 1:				23.6% ***				32.7% ***
Gender	−3.08	2.75	−0.077		−2.01	2.499	−0.044	
Child’s Age	0.036	0.945	0.003		−0.116	0.821	−0.008	
Gender of Parent reporting	−0.787	2.910	−0.019		2.317	2.827	0.47	
Mother’s age	0.092	0.413	0.029		−0.328	0.380	−0.095	
Father’s age	0.396	0.298	0.164		0.449	0.276	0.161	
Father’s education years	−0.371	0.416	−0.110		−0.561	0.418	−0.161	
Mother’s education years	−0.945	0.448	−0.261 *		−0.832	0.458	−0.224	
Number of family members	2.25	4.276	0.242		4.358	2.284	0.403	
Number of rooms in the house	−6.58	1.484	−0.351 **		−7.535	1.215	−0.366 ***	
Number of Siblings	−0.431	4.248	−0.047		−2.245	2.322	−0.208	
Step 2:				4.7% **				7.7% ***
Gender	−1.61	2.71	−0.040		−0.681	2.368	−0.015	
Child’s Age	0.180	0.919	0.015		0.062	0.774	0.004	
Gender of Parent reporting	−0.648	2.82	−0.016		2.233	2.666	0.045	
Mother’s age	0.115	0.401	0.036		−0.137	0.360	−0.040	
Father’s age	0.394	0.290	0.163		0.384	0.261	0.138	
Father’s education years	−0.335	0.405	−0.099		−0.472	0.394	−0.135	
Mother’s education years	−1.00	0.435	−0.277 *		−0.934	0.433	−0.252 *	
Number of family members	0.367	4.193	0.039		2.946	2.169	0.273	
Number of rooms in the house	−5.99	1.453	−0.320 **		−6.596	1.158	−0.320 ***	
Number of siblings	1.11	4.153	0.120		−1.364	2.195	−0.126	
Type of surgery (Elective/Emergency)	9.946	2.987	0.226 **		14.208	2.607	0.289 ***	
Step 3:				2.1% *				2.5% **
Gender	−1.19	2.68	−0.030		−0.462	2.324	−0.010	
Child’s Age	0.248	0.909	0.020		0.056	0.760	0.004	
Gender of Parent reporting	−0.392	2.79	−0.009		2.605	2.618	0.053	
Mother’s age	0.114	0.397	0.036		−0.139	0.353	−0.040	
Father’s age	0.389	0.286	0.161		0.374	0.256	0.134	
Father’s education years	−0.469	0.404	−0.139		−0.631	0.390	−0.181	
Mother’s education years	−0.966	0.430	−0.267 *		−0.880	0.425	−0.237 *	
Number of family members	−0.310	4.153	−0.033		2.058	2.146	0.191	
Number of rooms in the house	−5.93	1.436	−0.316 **		−6.282	1.140	−0.305 ***	
Number of siblings	1.57	4.108	0.170		−0.755	2.162	−0.070	
Type of surgery (Elective/Emergency)	7.89	3.086	0.179 **		11.297	2.717	0.230 ***	
Parent’s subjective feeling of being prepared	−7.17	3.132	−0.162 *		−9.046	2.859	−0.178 **	
R^2^ Total	30.4% *				43% **			

*** *p* < 0.001 ** *p* < 0.01 * *p* < 0.05.

**Table 2:** Crosstabs for the associations between the parents’ feeling of preparation and the children’s status according to cutoff score in the UCLA-PTSD and YCBC questionnaires.

	UCLA-PTSD			YCPC	
		Under Cutoff	Above Cutoff	Under Cutoff	Above Cutoff
Parents feeling prepared	Observed	166	16	170	13
	Expected	155.9	26.1	157.6	25.4
Parents feeling unprepared	Observed	49	20	47	22
	Expected	59.1	9.9	59.4	9.6
χ^2^	25.727 (*p* < 0.001)			9.343 (*p* < 0.001)	
eta	0.229			0.221	

## Data Availability

Data is contained within the article.
